# Proprioceptive Body Illusions Modulate the Visual Perception of Reaching Distance

**DOI:** 10.1371/journal.pone.0131087

**Published:** 2015-06-25

**Authors:** Agustin Petroni, M. Julia Carbajal, Mariano Sigman

**Affiliations:** 1 Departamento de Física, FCEN-UBA, Ciudad Universitaria, (1428) Buenos Aires, Argentina; 2 Universidad Torcuato Di Tella, Sáenz Valiente 1010, (1428) Buenos Aires, Argentina; Birkbeck, University of London, UNITED KINGDOM

## Abstract

The neurobiology of reaching has been extensively studied in human and non-human primates. However, the mechanisms that allow a subject to decide—without engaging in explicit action—whether an object is reachable are not fully understood. Some studies conclude that decisions near the reach limit depend on motor simulations of the reaching movement. Others have shown that the body schema plays a role in explicit and implicit distance estimation, especially after motor practice with a tool. In this study we evaluate the causal role of multisensory body representations in the perception of reachable space. We reasoned that if body schema is used to estimate reach, an illusion of the finger size induced by proprioceptive stimulation should propagate to the perception of reaching distances. To test this hypothesis we induced a proprioceptive illusion of extension or shrinkage of the right index finger while participants judged a series of LEDs as reachable or non-reachable without actual movement. Our results show that reach distance estimation depends on the illusory perceived size of the finger: illusory elongation produced a shift of reaching distance away from the body whereas illusory shrinkage produced the opposite effect. Combining these results with previous findings, we suggest that deciding if a target is reachable requires an integration of body inputs in high order multisensory parietal areas that engage in movement simulations through connections with frontal premotor areas.

## Introduction

Reaching an object with our hand seems easy, effortless and straightforward. Nevertheless, it represents a computational challenge since the brain must combine body and object reference frames to accomplish the task. Although the neurobiology of reaching has been extensively studied in human and non-human primates (see reviews by [1, 2]), the mechanisms that allow a subject to decide -without engaging in explicit action- whether an object is reachable remain debated. Recent TMS studies showed that motor areas are necessary in reaching decision tasks [3, 4], suggesting that judging what is reachable near the reach limit relies on mental simulations of the specific reaching movement. This idea was supported by behavioral experiments showing that reach distance estimation [5, 6] (see also [7]) and long range distance estimation [8] are affected when the cost of the motor output is manipulated (e.g. by adding weight to the arm). Similarly, it has been shown that imagined and actual reaching movements share broad brain regions, mainly dorsal premotor and posterior parietal cortex (PPC, [9–11]). The PPC integrates different sensory modalities (inputs) such as visual and proprioceptive information from the object and the body respectively, and combine them to generate a motor command downstream in motor regions [12–16]. This first integration conforms the body schema -an implicit multisensory representation of the body- which is distinct, although related to the explicit body representation or body sense [17–20]. The space near the body and within reach (peripersonal space) is closely linked to the body schema: Non-human primates studies described multimodal neurons in the premotor and the PPC with tactile receptive fields (e.g. from the hand) overlapping with adjacent auditory or visual receptive fields. When the animal moved its body part, the auditory or visual fields accompanied the tactile receptive field, representing the visual or auditory space centered on that body part [21–27]. An interesting property of the peripersonal space is its dynamic change with learning. Iriki and colleagues [28] showed in monkeys that the hand-centered visual receptive fields of a group of parietal neurons elongate after using a tool to reach food. Analogously, tool use has been shown to reshape body schema and peripersonal space in humans [6, 29–33]. For instance, Witt and colleagues demonstrated that holding a tool alters perceived distances, but only when subjects intended to use it [34]. In the same vein, Longo and Lourenco found a correlation between individual arm length and the representation of near space [35], supporting the notion that near-space perception relies on an intrinsic body metric. Taken together, this evidence suggests that body schema plays an important role in the estimation of reaching distances. However, the mentioned manipulations are more functional than perceptual, given that they imply motor practice with a tool. If a multisensory implicit body representation is mediating reaching distance estimation, on-line body size illusions should affect reaching judgments as well. Following this rationale, here we hypothesize that inducing proprioceptive illusions of finger size will alter the estimation of reachable distances. To test this hypothesis we induced a proprioceptive illusion of extension or shrinkage of the right index finger [36] while participants judged a series of LEDs as reachable or non-reachable without producing any actual movement. Our effort can be seen in analogy to other studies which have shown—in different domains—that illusory changes in our body size influence the size of visual or tactile objects [37, 38]. Our results support previous studies showing that reaching decisions near the reach limit depend dynamically on the body schema.

## Methods

### Participants

23 subjects from the campus of Buenos Aires University, 16 females (22.9 ± 3.3 years old, mean ± SE) participated in the study after giving written informed consent. They did not present any neurological or psychiatric disorders. The experimental procedure was approved by the Centro de Educación Médica e Investigaciones Clínicas “Norberto Quirno” (CEMIC)’s Ethical Committee and carried out according to the Declaration of Helsinki.

### Setup

**Fig 1:**
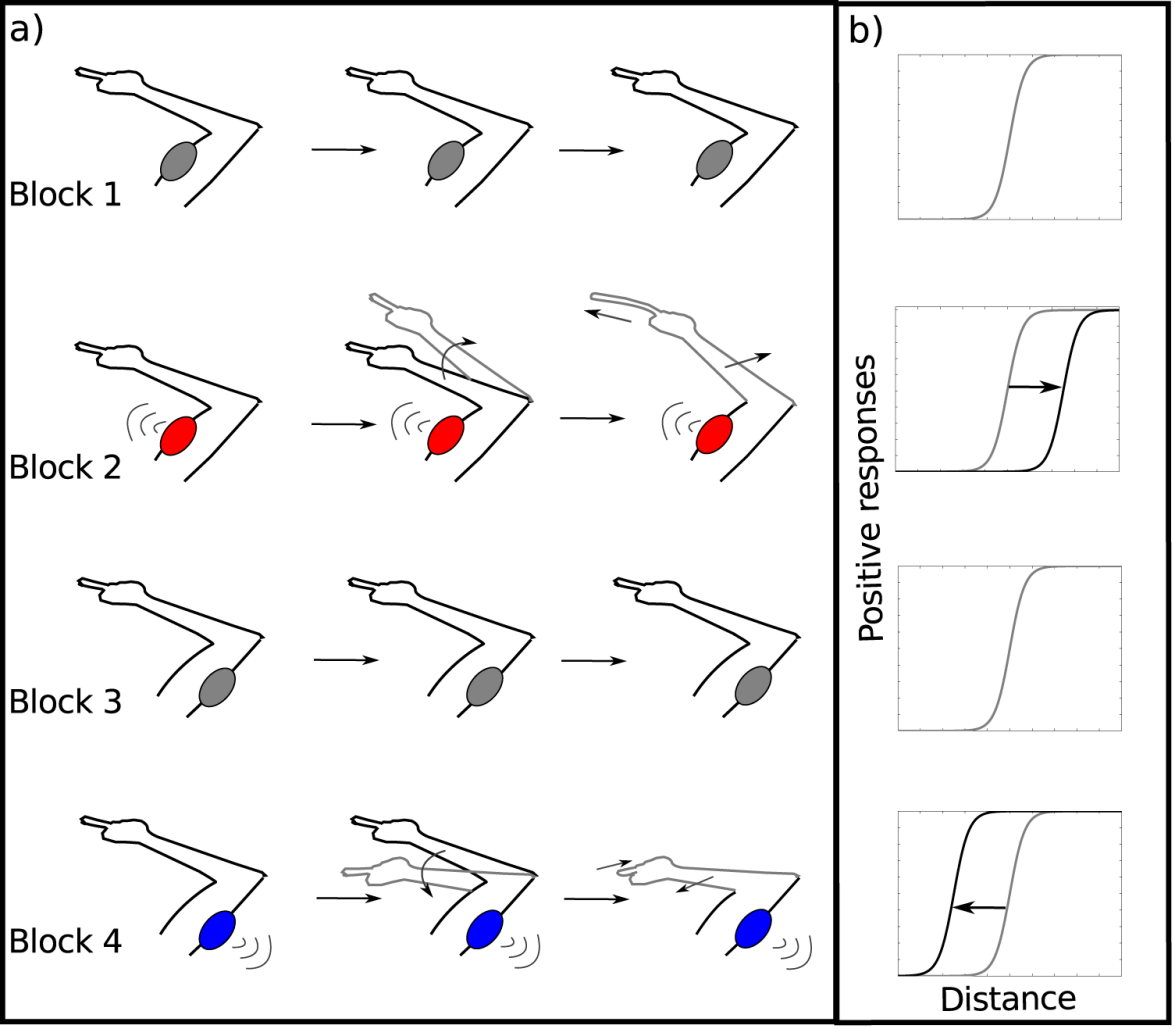
Experimental Design and predictions. (a) In the firsts two blocks the stimulator was placed in the biceps muscle but it only vibrates in the second block (depicted in red). In the following two blocks the protocol was identical, except that the stimulator was placed in the triceps muscle (blue). Blocks 1 and 3 are used as baselines for blocks 2 and 4, respectively. (b) The model predicted that during the finger extension (shrinkage) illusion, there is a right (left) shift in the psychophysics curve, corresponding to an increase (decrease) in reaching distance perception.

Participants sat in front of a horizontal table with a row of 21 red LEDs spaced 1 centimeter apart and aligned with the subject’s sagittal plane. The distance between the eyes and the table was kept constant with a chinrest and the entire experiment was completed in total darkness to avoid external visual references other than the LEDs [39, 40]. In order to induce an illusory change in finger size, participants placed their arms below the table and beneath their legs in a relaxed position, without contact with these objects. The tip of the right index was held firmly with the left index and thumb. A mechanical device vibrating at 75 Hz was placed on the biceps or triceps muscle inducing an arm extension or flexion kinesthetic illusion, respectively. When subjects hold their right index with their left hand, some of them experience an elongation or shrinkage of the right index finger (see Fig 1) [36, 38].

### Procedure and task

Each trial began with one randomly selected LED light turned on. Subjects were instructed to judge whether or not they could reach the target LED with their right hand, without performing the actual movement. The light turned off when they responded by pressing a pedal with their left or right foot indicating yes or no, respectively. Thereafter, a new trial started with a fixed inter-trial interval of 1.2 seconds. In each block of trials, all LEDs were sampled without consecutive repetitions in a pseudorandom order. The series of 21 reaching positions was repeated 5 times shuffling each time, thus completing a total of 105 trials per block. Subjects’ responses and their respective response times (RT, i.e. the time elapsed since a LED was turned on to the moment the subject pressed the pedal) were collected with Matlab using the Psychophysics Toolbox extension [41]. The complete experiment began with a short training period to familiarize the subject with the task followed by four experimental blocks (see Fig 1). In the first two blocks the stimulator was placed over the biceps muscle but it was only activated during the second block. As previously mentioned, this stimulation was expected to induce an illusory elongation in some, but not all, of the subjects. Then, in the following two blocks the protocol was identical except that the stimulator was placed over the triceps muscle and only turned on during the fourth block, which was meant to induce an illusory shrinkage. Blocks 1 and 3 (stimulation off) served as baselines for blocks 2 and 4 (stimulation on), respectively (Fig 1). Volunteers never performed the actual reaching movement. After the experiment, participants reported whether they had felt an illusory size change in blocks 2 and 4. Subjects who reported to feel the illusion were then asked to indicate the perceived change in size placing the left index finger where they felt the illusory tip of the distorted right finger. The experimenter measured this quantitative report of the illusion (RI) with a ruler.

### Calibration

Before the training session, the central LED was turned on in total darkness and subjects were asked to respond verbally one of three options: the light is reachable, non-reachable, or it is in the reach limit. Depending on the response, the LED was switched off and the table was moved, the LED turned on again, iterating until the subject declared that the light was in the limit of reaching. Subsequently, the table was kept in this position during the rest of the experiment to sample the response curve centered in the central LED (see Fig 1).

### Data Analysis

**Fig 2:**
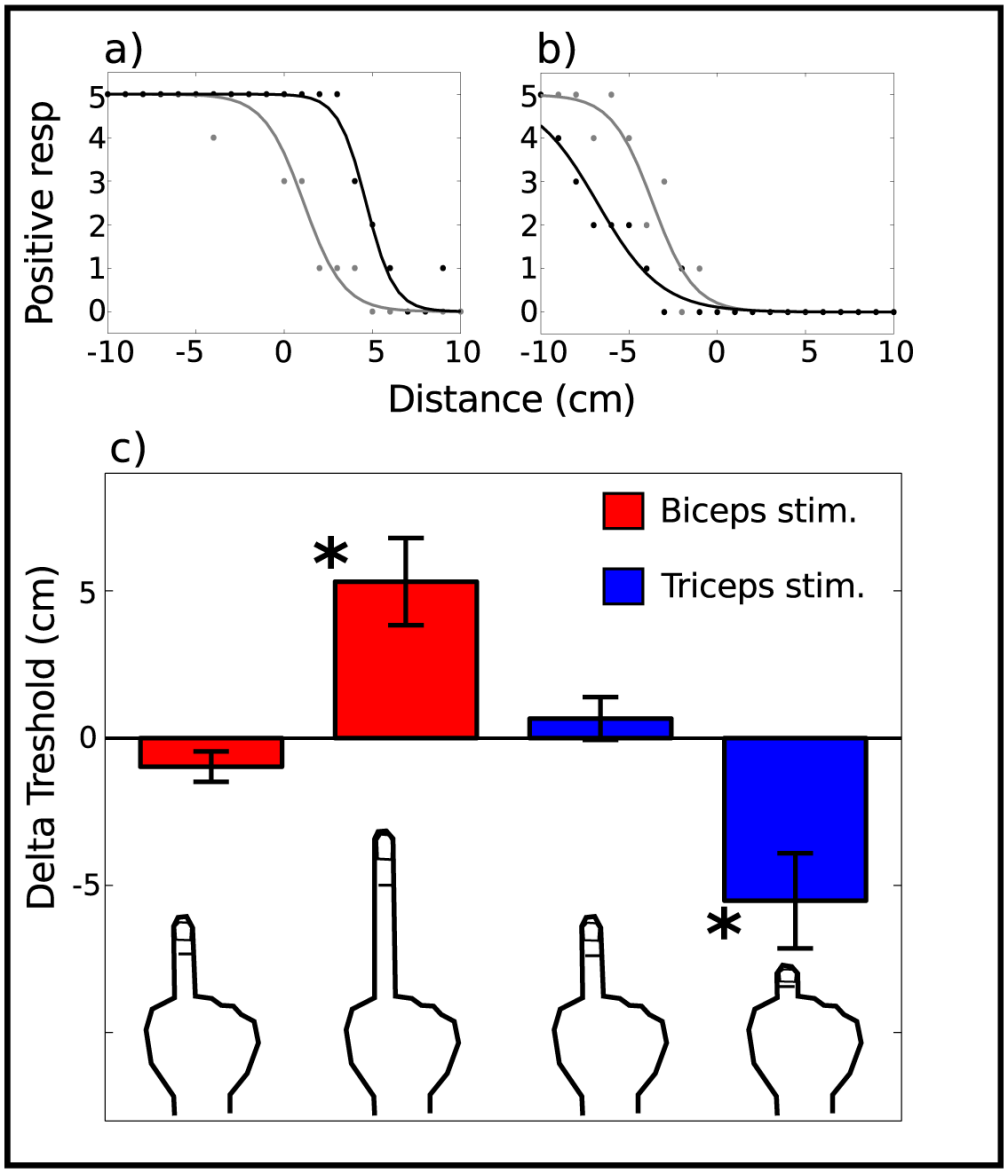
Reaching curves of a representative subject that felt both illusions and group results. (a) Block 1 (baseline, gray) and block 2 (extension illusion, black). (b) Block 3 (baseline, gray) and block 4 (shrinking illusion, black). (c) Delta threshold (experimental minus baseline block) for both muscle stimulation protocols. Each color corresponds to a muscle, biceps is depicted in red, triceps in blue. For both muscles, the left columns represent subjects that did not experience the corresponding illusion, whereas the right columns represent subjects that reported the ilusion (*p < 0.005).

Subjects’ responses and response times were first analyzed individually for each of the four blocks. Within a block, positive responses were summed for each LED position giving a total of 21 data points per block and subject, with a maximum value of 5 (all positive responses) and a minimum of 0 (no positive responses). The accumulated positive responses *P* describe a psychometric curve as a function of the target position. Data points were fitted with a logistic function (Fig 2) based on a drift-diffusion model often used to describe 2AFC tasks [42] using the following equation,

$$\begin{array}{c} P\left(x\right)=\frac{5}{1+e^{-Ak(x-T)}} \end{array}$$ (1)

where *x* is the position of the target measured in cm from the central LED, *T* is the critical value of *x* at which the transition occurs, *A* represents a decision boundary, and k represents the sensitivity of the subject (these last two parameters are not analyzed in the present study). The parameter *T*, which indicates the transition position at which subjects begin to report more than 50% of the times that the target is reachable, is referred to as the *reaching threshold*. Reaching thresholds were calculated for each block of each individual subject. We then used block 1 (stimulation off) as a baseline of block 2 (stimulation on) and, analogously, block 3 as a baseline of block 4 (see Fig 1). Hence, we calculated the differences in thresholds between block 2 and block 1 for the elongation protocol and between block 4 and block 3 for the shrinkage protocol. These differences are referred to as *delta thresholds* (ΔT).

**Fig 3:**
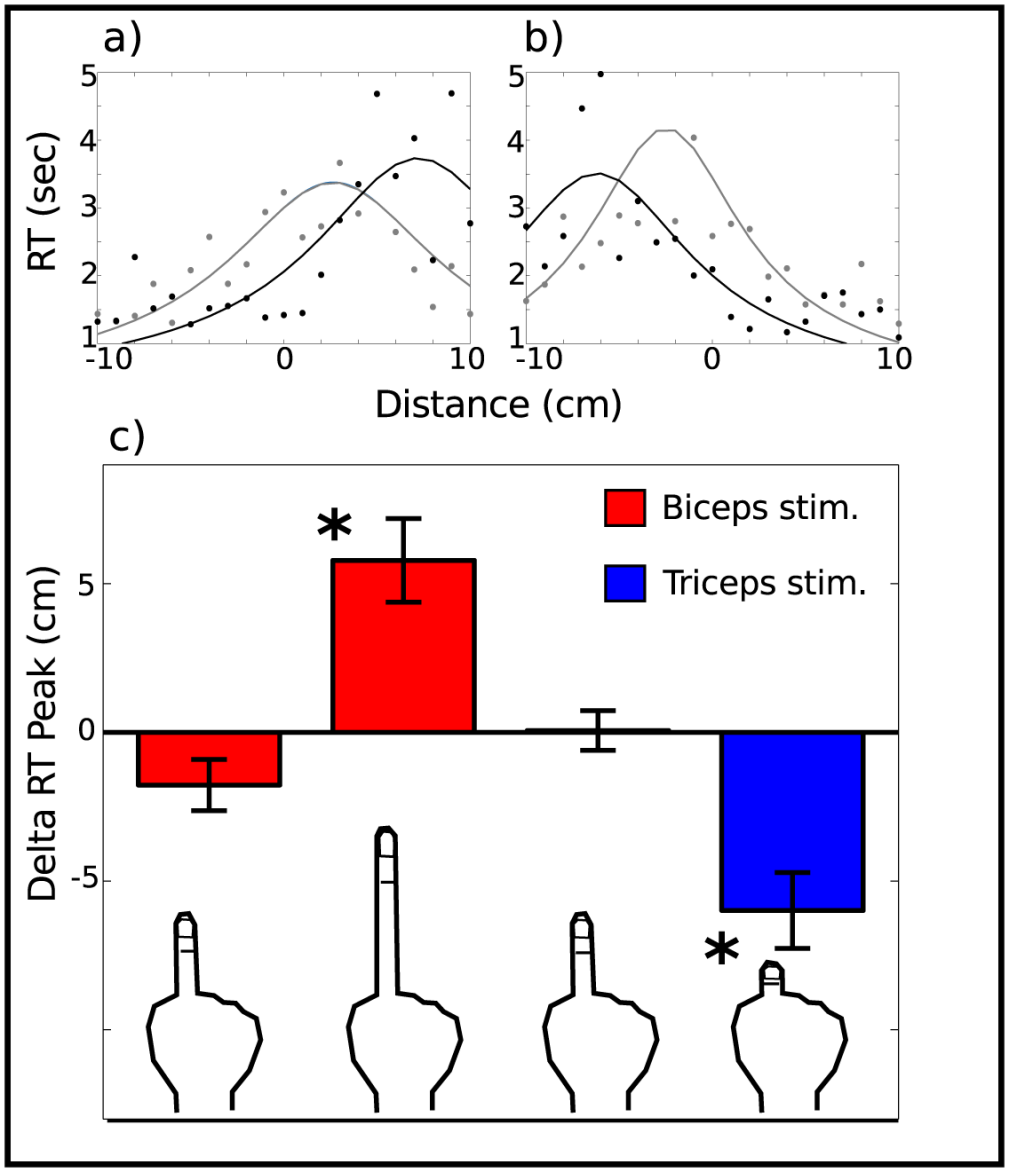
Reaction time vs distance for a representative subject that felt both illusions and RT group results. (a) Block 1 (baseline, gray) and block 2 extension illusion, black) reaction times. (b) Block 3 (baseline, gray) and block 4 (shrinking illusion, black) reaction times. Both curves were from the same subject shown in Fig 2a and 2b. (c) Spatial shift in RT peak (experimental minus baseline block) for both muscle stimulation protocols. Each color corresponds to a muscle, biceps is depicted in red, triceps in blue. For both muscles, the left columns represent subjects that did not experience the corresponding illusion, whereas the right columns represent subjects that reported the ilusion (*p < 0.005).

To analyze the response times, the mean of the RTs was calculated at each target position, giving a total of 21 data points per block and subject (see Fig 3). Individual trials with RT values above 10 s or below 200ms were discarded prior to analysis. The mean response times were fitted with the following equation, derived from the drift-diffusion model,

$$\begin{array}{c} \tau \left(x\right)=\frac{A}{k(x-T)}\tanh(Ak(x-T))+\tau_{R} \end{array}$$ (2)

where A, k, x and T are the same parameters described in Eq (1), and *τ*_*R*_ is the residual response time. Analogously to the response threshold, the position of the peak of the response times (T) was obtained for each block, as an implicit measure of reaching transitions. As with ΔT, ΔRT was calculated for each subject as the difference between the position of the response time peak of block 2 and block 1, or block 4 and block 3, for the extension and shrinkage conditions, respectively. To test for an effect of the proprioceptive illusion on reaching thresholds and response times, successive analyses were conducted on both stimulation sites (biceps and triceps). For each site, we compared the shifts ΔT and ΔRT of the subjects who reported the illusion to be present to those who reported the illusion to be absent. These statistical comparisons were carried out with Welch’s t-tests. Furthermore, regression analyses were used to test an association between subjects’ perceived finger size change reports (RI) and measured parameters (ΔT, ΔRT). These analyses were only performed when the illusion was present.

## Results

### Efficiency of the illusion paradigm and amplitude of the illusion: non-verbal final report

13 (56.5%) out of 23 participants reported experiencing the finger extension illusion and 9 (39.1%) reported the shrinkage illusion. 7 reported experiencing both illusions, 6 only extension, 2 only shrinkage and 8 none of them. The efficiency of the illusion paradigm is in agreement with a previous study that reported that triceps stimulation was less effective than biceps [38]. The average reported elongation was 9.7±1.5cm (mean ± SE) and the average reported shrinkage was −9.6±2.1cm.

### Changes in reaching threshold

We first evaluated the effect of the illusion on the reaching threshold. For this, we analyzed the shift of the threshold induced by the stimulation of the biceps and triceps depending on whether participants reported the illusion to be present or absent (see Fig 2). We subtracted the baseline threshold (block 1 or block 3) to the stimulation reaching threshold (block 2 or block 4, respectively), obtaining ΔT. Our working hypothesis is that the perceived illusion (and not muscle stimulations per se) affects perceived reach distance. Hence, we expected to find a significant difference in ΔT between illusion present vs illusion absent groups. The mean shifts of the reach threshold when the illusion was present were Δ*T*_2−1_ = 5.3 ± 1.5 cm (mean ± SE) after stimulation of the biceps, and Δ*T*_4−3_ = −5.5 ± 1.6 cm after stimulation of the triceps. On the other hand, the average shifts in absence of the illusion were Δ*T*_2−1_ = −1.0 ± 0.5 cm and Δ*T*_4−3_ = 0.7 ± 0.7 cm. Statistical analyses revealed a significant difference in ΔT when the subjects reported the illusion to be present compared to when the illusion was absent both in the biceps (*t* = 4.02, *df* = 14.8, *p* < 0.001) and the triceps (*t* = −3.49, *df* = 11.3, *p* < 0.005). Furthermore, the shift in reaching threshold when the illusion was absent was not significantly different from 0 in either stimulation site (biceps: *t* = −1.89, *df* = 9, *p* = 0.09; triceps: *t* = 0.92, *df* = 13, *p* = 0.38). The direction of the observed effect is consistent with the illusion, indicating that subjects reported reaching longer distances when feeling that their finger was extended, and shorter distances when feeling that their finger had shrunk, while these distances were not affected when the illusion was absent.

### Response Time peak shift

In binary choices, decision times increase when the signal is close to the decision boundary. It is thus expected that participants will respond more slowly when they have to judge whether a distance close to their reach limit is reachable or not. Hence, we predict that an implicit measure of the shift in the decision boundary should be revealed in the pattern of response times. To examine this hypothesis, we evaluated the effect of the illusion on the position of the peak of the response times. The mean shift of the RT peak position when the illusion was reported as present was Δ*RT*_2−1_ = 5.8±1.4 cm (mean ± SE) after stimulation of the biceps, and Δ*RT*_4−3_ = −6.0±1.3 cm after stimulation of the triceps. On the other hand, the average shifts in the absence of illusion were Δ*RT*_2−1_ = −1.8±0.9 cm and Δ*RT*_4−3_ = 0.1±0.7 cm, respectively (see Fig 3).

Statistical analyses revealed a significant difference in ΔRT when the subjects reported the illusion to be present compared to when the illusion was absent both in the biceps (*t* = 4.58, *df* = 19.0, *p* < 0.0005) and the triceps (*t* = −4.20, *df* = 12.4, *p* < 0.005). Similarly to ΔT, the shift when the illusion was absent was not significantly different from 0 in either stimulation site (biceps: *t* = −2.06, *df* = 9, *p* = 0.07; triceps: *t* = 0.09, *df* = 13, *p* = 0.92).

As with ΔT, the illusion induced a significant effect on the response times, shifting the peak positively when the subjects perceived an elongation of their finger and negatively when they perceived a shrinkage. Altogether these results suggest that the change in perceived finger size affected the perception of reaching distance.

### Regression analysis

**Fig 4:**
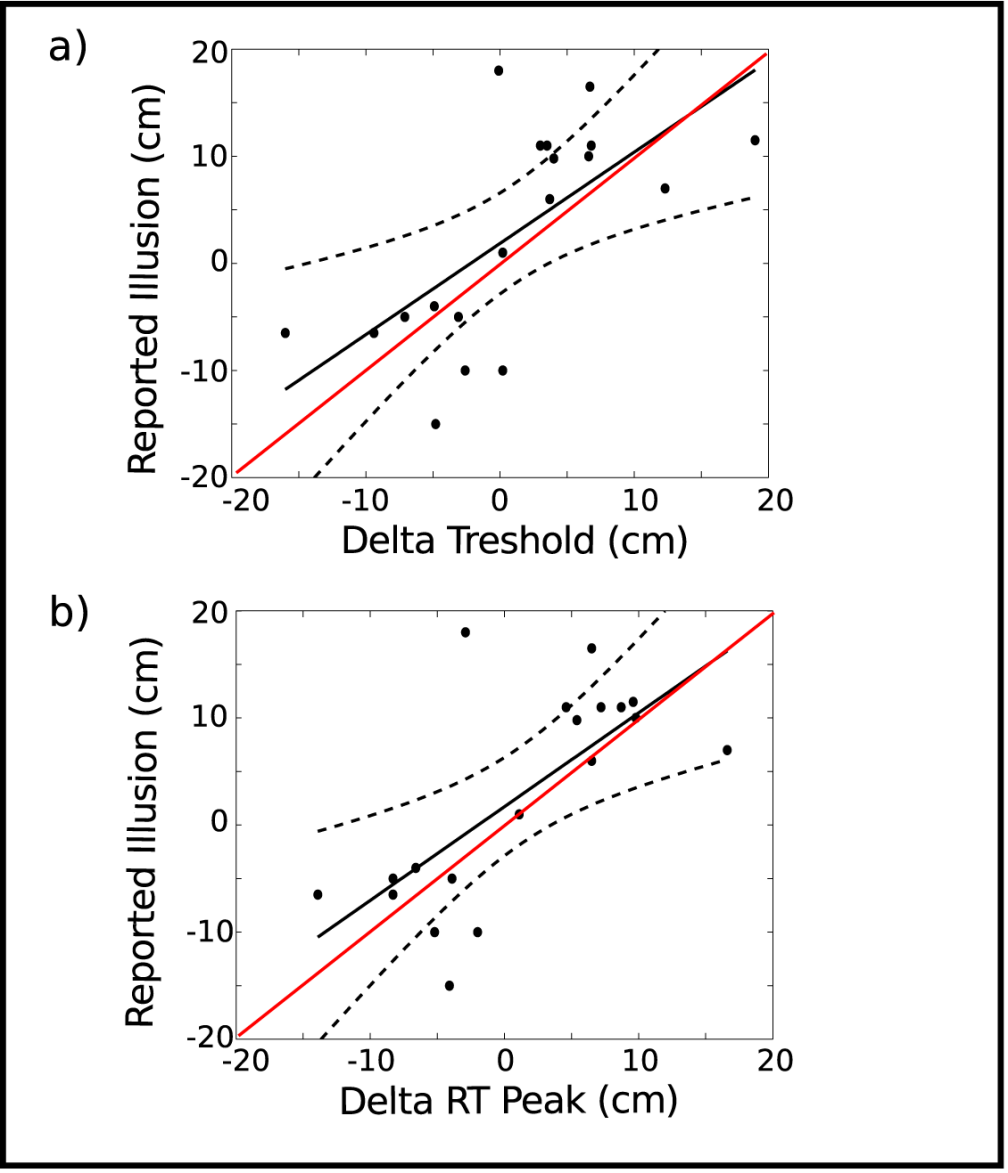
Finger size change report vs Delta Threshold and Delta RT. (a) Linear regression of subjective finger size change on reaching threshold shift. (b) Linear regression of subjective finger size change on reaction time peak shift. In both plots the regression line and 95% confidence intervals are depicted in black and dashed black lines, respectively. Identity line (y = x) is depicted in red.

If judgments of reach are based on body representations, those participants perceiving a greater extension of their reach region should also feel a greater illusion. Similarly, the RT distribution should also shift revealing greater RTs close to the displaced threshold. To examine these predictions, we performed a linear regression of the reported illusion RI(s) to ΔT(s) and ΔRT(s) where s indicates the measure for each specific subject (Fig 4). The regression of subjective illusion reports on T yielded a significant slope *β*_Δ*T*_ = 0.85 (p < 0.005, f = 14.26, df = 18). The regression of subjective illusion reports on RT yielded a significant slope *β*_Δ*RT*_ = 0.88 (p < 0.001, f = 16.12, df = 18). Both regression slopes overlap with the identity line within the 95% confidence interval of the regression slope. Thus, increases in the estimated reach threshold result in proportional -with a proportion coefficient not different from 1- increases in the perceived illusion.

## Discussion

The aim of this study was to evaluate the causal role of multisensory body representations in the perception of reachable space. We reasoned that if body representations are used to estimate reach, an illusion of the finger size induced by proprioceptive stimulation should propagate to the perception of reaching distances. In particular, during the finger extension illusion we expected a shift in the psychometric curve corresponding to an increase in reaching distance perception, whereas the opposite was expected for the shrinkage illusion (Fig 1b). We found that the finger size illusion indeed shifts the reaching threshold towards the expected direction in both illusion types. Secondly, response time distribution varied accordingly, revealing longer decision times near the shifted threshold. Finally, both reaction times and explicit reaching judgments are good predictors of the extent of the illusion reported after the experiment. These findings confirm our hypothesis. The last two findings suggest that both explicit (reflected by ΔT) and implicit (reflected by ΔRT) body representations might be used to estimate reaching distances. In other words, the induced illusions affected the body schema and also the conscious representation of the body, also referred to as body image [18]. Our findings are in line with a study that showed an effect of peripersonal space expansion in explicit reaching judgments [34]. Furthermore, the symmetrical effects on reaching estimation are in agreement with previous reports of both extension [20] and shrinkage [7] of the peripersonal space. The body size illusions we induced by stimulating proprioceptors affected the body representation, but it is worth noting that this is different from the body representations underlying proprioception described in other studies [17]. In this study the peripersonal space, defined by the body schema, was measured with decision thresholds and reaction times related to a potential reaching movement. It would be important to assess in future experiments whether the proprioceptive illusion affects peripersonal space also as a sensory space, using the detection of approaching visuo-tactile, auditory-tactile or visuo-auditory stimuli interaction tasks [20]. In this way, it would be possible to determine if the body schema as a general concept is affected, both as a motor and as a sensorial body representation. Proprioception informs about the movement and position of body parts. It is required for visually guided action because it specifies the position of the eyes relative to the head, the position of the head relative to the body and the relative position of body parts [39]. A direct evidence of the importance of proprioception in human reaching has been illustrated in a recent experiment in which neck muscles proprioceptors were stimulated during a visually guided reaching task, showing that pointing movements are altered in the presence of head movement illusions [43]. Some recent studies have shown that body size visual illusions modulate the size of perceived visual objects [37]. Likewise, illusory shrinkage and growth of visual objects using minifying or magnifying goggles, respectively, alter the perception of object size but this effect diminished when the subject’s hand was included in the visual scene, suggesting that our body acts as a perceptual ruler [44]. Regarding the possible neural substrates that may be responsible for this effect, previous studies showed that body size illusions induced by proprioceptive stimulation are mediated by hierarchically high-order somatosensory areas in the parietal cortex [45]. Human reaching areas conform a frontoparietal network where parietal areas integrate multisensory body information and vision, processing spatial coordinates of body and object, respectively [9]. In turn, these areas are connected to frontal areas, mainly the dorsal premotor cortex to execute reaching movements with corresponding motor commands [46–48]. Finally, experiments in non-human primates showed that parietal reaching areas activate during reaching decisions as well as prior to reaching decisions, indicating that they also represent potential reaches [10, 49]. Summing up, we speculate that deciding if a target is reachable requires an integration of body inputs in high order multisensory parietal areas that engage in movement simulations through connections with frontal premotor areas. A very important aspect of our experimental design is that the experiment was conducted in total darkness with low intensity LEDs to avoid contaminating light. It has been established that the parietal cortex assigns weights to each unisensory signal to integrate a multisensory percept, realigning those inputs with less weight [50]. Our big effect of proprioception on vision might be caused by the position uncertainty of our visual stimuli in the absence of other visual references. Otherwise, the effects may have been modest, or wiped out by visual predominance [51]. In the same vein, our big effect of proprioceptive illusions on peripersonal space perception suggested that this type of illusion can be implemented in virtual reality environments. In fact, mechanical vibration devices are low cost, small sized and light weighted. Multiple vibrators placed in different muscles can be used in combination with visual stimulation to enhance virtual reality vividness. To conclude, our findings propose causal experimental evidence indicating that reaching decisions near the peripersonal space entail a mental simulation relying on body representations which are likely being used as input for motor simulations.

## Supporting Information

S1 Data Raw data file. (CSV)
